# Flexible Piezoelectric Energy Harvesting from Mouse Click Motions

**DOI:** 10.3390/s16071045

**Published:** 2016-07-06

**Authors:** Youngsu Cha, Jin Hong, Jaemin Lee, Jung-Min Park, Keehoon Kim

**Affiliations:** 1 Center for Robotics Research, Korea Institute of Science and Technology, Seoul 02792, Korea; hoji0207@kist.re.kr (J.H.); jmlee87@kist.re.kr (J.L.); pjm@kist.re.kr (J.-M.P.); khk@kist.re.kr (K.K.); 2 School of Mechanical Engineering, Korea University, Seoul 02841, Korea

**Keywords:** energy harvesting, mouse click motion, piezoelectric material

## Abstract

In this paper, we study energy harvesting from the mouse click motions of a robot finger and a human index finger using a piezoelectric material. The feasibility of energy harvesting from mouse click motions is experimentally and theoretically assessed. The fingers wear a glove with a pocket for including the piezoelectric material. We model the energy harvesting system through the inverse kinematic framework of parallel joints in a finger and the electromechanical coupling equations of the piezoelectric material. The model is validated through energy harvesting experiments in the robot and human fingers with the systematically varying load resistance. We find that energy harvesting is maximized at the matched load resistance to the impedance of the piezoelectric material, and the harvested energy level is tens of nJ.

## 1. Introduction

Recent advancements and developments in the area of wearable devices have stimulated the demand for energy harvesting system [1,2,3]. Various energy sources, such as heat [4] and motions [5,6,7], in human body can be converted into useful electric energy by using energy transducers. In this context, energy harvesting to support the power of wearable devices using the human body energy sources can offer several benefits: permanent lifetime and weight reduction through the needlessness of batteries [8].

Piezoelectric materials are a good solution as energy transducer [9,10]. Especially, Lead zirconate titanate (PZT) [11,12,13], Polyvinylidene fluoride (PVDF) [14,15,16,17], Macro-fiber composite (MFC) [18,19,20,21], Aluminum nitride (AlN) [22], and Zinc oxide (ZnO) [23] have been used as typical energy transducers. They convert the kinetic energy of surrounding environment into electric energy. For example, energy harvesting from PVDF when subjected to various wind speeds and water droplets has been evaluated in [14]. The feasibility of energy generation of PVDF cantilever with a magnetic mass has been experimentally studied in [15]. PVDF is the most flexible piezoelectric material among them although it has the lowest electromechanical coupling [24,25]. The flexibility can offer important advantage in the development of wearable devices by reducing the inconvenience of wearing.

Here, we theoretically and experimentally study energy harvesting using the flexible energy transducer, PVDF, attached to robot and human index fingers during mouse click motions. The ability of harvesting energy from mouse click motions may be applicable to the development of self-powered computer mouse [26], wearable mouse glove [27], or hand motion recognition device [28]. Various researches about energy harvesting using human motions have been reported. For instance, energy harvesting from foot strike during human walking by using shoes including PZT and PVDF has been introduced in [5]. In [29], human walking motion has been also used for energy harvesting by using a backpack instrumented with piezoelectric shoulder straps. In [30], a device using plucked piezoelectric bimorphs for energy harvesting from knee motions during human walking has been reported. Energy harvesting on human limb motions has been demonstrated in [6]. In [7], energy harvesting using the jaw movements of human through the piezoelectric chin strap has been demonstrated. Energy harvesting from two different piezoelectric transducers attached to the human body for five human activities has been studied in [31]. Interestingly, a glove to harvest electric energy from the kinetic energy of the fingers has been reported in [32]. Therein, four couples of piezoelectric transducers have been integrated into the glove in correspondence with the fingers joints, but the finger motions for energy harvesting have not been specified. Moreover, ZnO based energy harvester using stretched and released states of index finger has been demonstrated in [33]. In this work, we focus on the mouse click motions of fingers for energy harvesting. Specifically, we model the mouse click motions and the energy transducing of the piezoelectric material. The proposed model is validated through experiments using robot and human index fingers. The energy transducer, PVDF, is attached to fingers by wearing a glove with a pocket for piezoelectric material. In the robot finger, we can reduce the effect by the variation of the human motion, and quantitatively analyze the energy harvesting from the mouse click motions. From a practical point of view, this work addresses the untapped research question of energy harvesting from mouse click motions. From a methodological point of view, the main contributions of this effort are: (i) developing an electromechanical model to study energy harvesting of flexible piezoelectric materials bent by mouse click motions; (ii) performing a thorough experimental campaign to validate the proposed modeling framework in various conditions, whereby one and double click motions, human and robot fingers, and shunting load resistance; and (iii) conducting a systematic analysis of its energy harvesting capacity.

This paper is organized as follows. In Section 2, we introduce the proposed modeling framework, including the inverse kinematic framework of parallel joints in a finger and the electromechanical coupling for the energy transducer. In Section 3, we describe the experimental scheme developed to study energy harvesting from the mouse click motions. In Section 4, we report and discuss the experimental results toward the validation of the model and the analysis of harvested energy from the mouse click motions of a robot finger and a human index finger. Conclusions are summarized in Section 5.

## 2. Modeling

### 2.1. Finger Model

**Figure 1:**
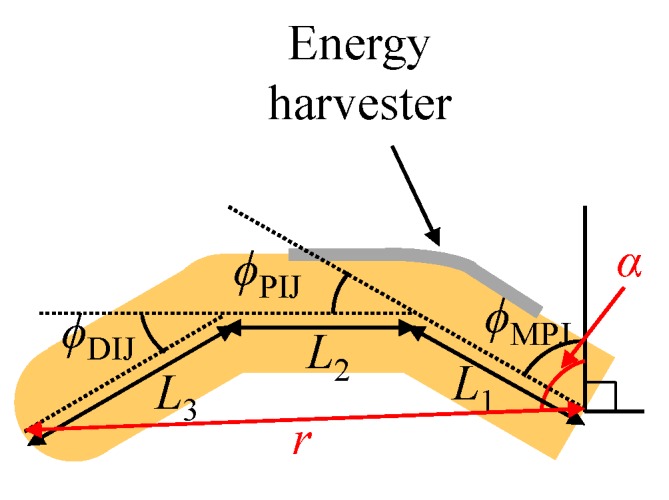
Schematic of parallel joints on finger.

The formulation of the model for index finger motion follows the inverse kinematic framework of parallel joints presented in [34] for studying human hand movements. Therein, the finger can be considered locally a three degree of freedom mechanism with three links in a plane, see Figure 1. The origin of the coordinate system lies at the index metacarpophalangeal (MP) joint. The angle of rotation of the MP joint is defined as $\phi_{\mathrm{MPJ}}$. The angles of the other two joints are labeled as $\phi_{\mathrm{PIJ}}$ (proximal interphalangeal (PI) joint) and $\phi_{\mathrm{DIJ}}$ (distal interphalangeal (DI) joint) [35]. The lengths of the three links are $L_{1}$ (proximal phalanx), $L_{2}$ (middle phalanx), and $L_{3}$ (distal phalanx) [35]. Moreover, we denote *r* and *α* with the distance between the tip and the origin of the finger and the angle between *r* and the starting position of the MP joint ($\phi_{\mathrm{MPJ}}=0$), respectively.

Following [34], the relationship among the lengths and distance is given by

(1) $$\begin{array}{c} \left(L_{1}^{2}+L_{2}^{2}+L_{3}^{2}-r^{2}-2L_{1}L_{2}-2L_{1}L_{3}+2L_{2}L_{3}\right)g^{10}\\ +\left(5L_{1}^{2}+5L_{2}^{2}+5L_{3}^{2}-5r^{2}+26L_{1}L_{2}+90L_{1}L_{3}-6L_{2}L_{3}\right)g^{8}\\ +\left(10L_{1}^{2}+10L_{2}^{2}+10L_{3}^{2}-10r^{2}+28L_{1}L_{2}-420L_{1}L_{3}-28L_{2}L_{3}\right)g^{6}\\ +\left(10L_{1}^{2}+10L_{2}^{2}+10L_{3}^{2}-10r^{2}-28L_{1}L_{2}+420L_{1}L_{3}-28L_{2}L_{3}\right)g^{4}\\ +\left(5L_{1}^{2}+5L_{2}^{2}+5L_{3}^{2}-5r^{2}-26L_{1}L_{2}-90L_{1}L_{3}-6L_{2}L_{3}\right)g^{2}\\ +(L_{1}^{2}+L_{2}^{2}+L_{3}^{2}-r^{2}+2L_{1}L_{2}+2L_{1}L_{3}+2L_{2}L_{3})=0 \end{array}$$

where *g* is an unknown variable related with the rotation angles. When we know $L_{1}$, $L_{2}$, $L_{3}$, and *r*, Equation (1) can be solved with at least two real solutions for *g*. Herein, we use the built-in function ‘Solve’ in MATHEMATICA (www.wolfram.com) to solve it. Among the solutions, the only valid solution in a reachable position is the positive real one. From the solution *g*, we obtain [34]

(2) $$\phi_{\mathrm{MPJ}}=\alpha -\arccos\left(\frac{s^{2}-r^{2}-L_{1}^{2}}{2rL_{1}}\right)$$

(3) $$\phi_{\mathrm{PIJ}}=6\arctan\left(g\right)$$

(4) $$\phi_{\mathrm{DIJ}}=4\arctan\left(g\right)$$

where $s=\sqrt{L_{2}^{2}+L_{3}^{2}-2L_{2}L_{3}\cos\left(\pi -\phi_{\mathrm{DIJ}}\right)}$.

We comment that the variations of the angles can be obtained through Equations (1)–(4) and the distance *r* changed by mouse click motions, without directly measuring the angles. In particular, we note that $\phi_{\mathrm{PIJ}}$ and $\phi_{\mathrm{DIJ}}$ are related to only the solution *g*.

### 2.2. Energy Harvesting Model

An energy harvester using a piezoelectric material is attached to the PI joint. When we assume the energy harvester has a beam structure, its electrical response is described by a relationship between the charge stored in the structure and the rotation of the beam as [11,20,36]

(5) $$Q\left(t\right)=CV\left(t\right)+\theta \tan(\phi_{\mathrm{PIJ}}\left(t\right))$$

where *Q* is the stored charge, *C* is the internal capacitance of the piezoelectric material, *V* is the voltage between the both electrodes of the piezoelectric material, *θ* is the electromechanical coupling coefficient, and *t* is the time variable.

When the piezoelectric material is open-circuited (Q=0), the voltage is reduced as

(6) $$V_{\mathrm{oc}}\left(t\right)=-\frac{\theta \tan(\phi_{\mathrm{PIJ}}\left(t\right))}{C}$$

Moreover, when a load resistor $R_{\mathrm{load}}$ is shunted to the piezoelectric material, the load voltage $V_{\mathrm{load}}$ is given by Ohm’s law, that is,

(7) $$\frac{dQ(t)}{dt}=-\frac{V_{\mathrm{load}}\left(t\right)}{R_{\mathrm{load}}}$$

By combining Equations (5) and (7), we derive

(8) $$C\frac{dV_{\mathrm{load}}\left(t\right)}{dt}+\theta \frac{d\tan(\phi_{\mathrm{PIJ}}\left(t\right))}{dt}=-\frac{V_{\mathrm{load}}\left(t\right)}{R_{\mathrm{load}}}$$

By numerically solving Equation (8) with the given $\phi_{\mathrm{PIJ}}\left(t\right)$ using MATHEMATICA by the built-in function ‘NDSolve’, we obtain the load voltage.

Additionally, we can calculate the energy transferred to the load resistor $E_{\mathrm{load}}$ from the piezoelectric material during t=T, following

(9) $$E_{\mathrm{load}}=\int_{0}^{T}\frac{V_{\mathrm{load}}^{2}\left(t\right)}{R_{\mathrm{load}}}dt$$

The integral is numerically computed using hundreds of Gaussian quadrature points at the selected cases to reduce the computational time.

## 3. Experiments

### 3.1. Experimental Setup

**Figure 2:**
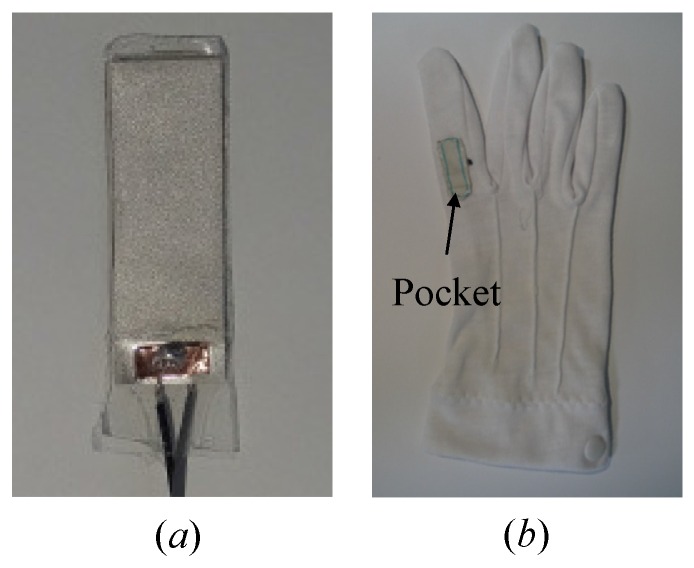
Pictures of (**a**) the piezoelectric composite and (**b**) the glove with the pocket.

The energy harvester has the unimorph beam structure to consist of PVDF as a piezoelectric material and mylar as a substrate, see a similar example in [16]. Specifically, a PVDF produced by Measurement Specialties (www.meas-spec.com) is glued on a thin mylar using epoxy 3 M DP460. The dimensions of the PVDF and mylar layers are $28\times 8\times 0.028\;{\mathrm{mm}}^{3}$ and $25\times 8\times 0.1\;{\mathrm{mm}}^{3}$, respectively. The PVDF surface is slightly bigger due to the connection with electrodes. Conductive adhesive 3 M copper foil tape 1181 is used for the both side electrodes. The overall structure is sealed with 3 M Scotch tape. Figure 2a displays the top view of the energy harvester used in this study.

**Figure 3:**
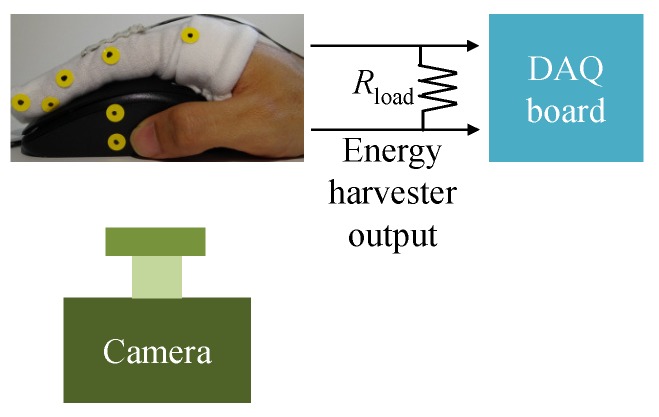
Experimental setup for a human finger.

To attach the energy harvester to a finger, we use a reform glove with a pocket, see Figure 2b. The experimental setup using a human finger is displayed in Figure 3. The human index finger has the size of $L_{1}=41\;\mathrm{mm}$, $L_{2}=22\;\mathrm{mm}$, and $L_{3}=20\;\mathrm{mm}$. All experiments are conducted on Logitech M-U0026 mouse. SONY FDR-AX30 camera is utilized to acquire the finger movements, and Xcitex Proanalyst software (www.xcitex.com) is used to track the selected markers on the finger. The resolution of the camera is set to 1920×1080 pixels, and the recording rate is 60frame/s. The recoding camera image scale is approximately 0.01cm/pixel in the experiments.

The output voltage from the piezoelectric material is acquired using National Instrument data acquisition (DAQ) board 6343 and a custom-made code in Labview (www.ni.com/labview). The input impedance of the DAQ board is 10GΩ in parallel with 100pF, and the sampling rate is 2000Hz. During experiments, the piezoelectric material is open-circuited or shunted with a load resistance varied from 1 to 99MΩ by using IET LABS RS-201W resistance decade box. A MATLAB (www.mathworks.com) script is utilized to remove the power source noise from the output voltage through a 2-order low pass Butterworth filter at 30Hz. The capacitance of the piezoelectric material, C=1.58nF, is measured using FLUKE-17B+ digital multimeter.

### 3.2. Robot Finger

**Figure 4:**
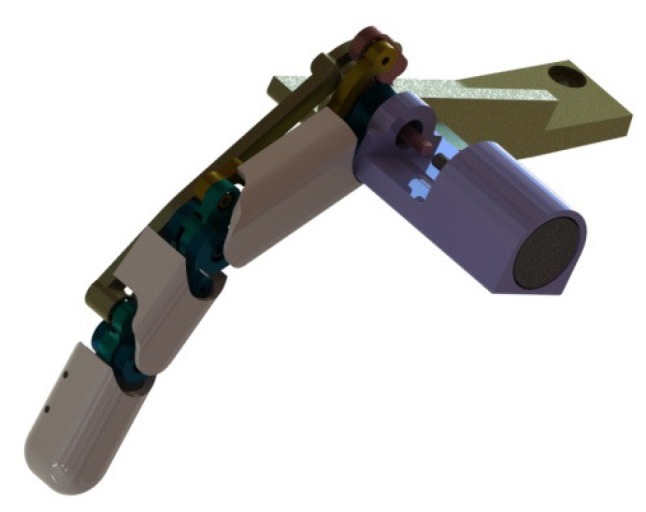
CAD model of a robot finger.

**Figure 5:**
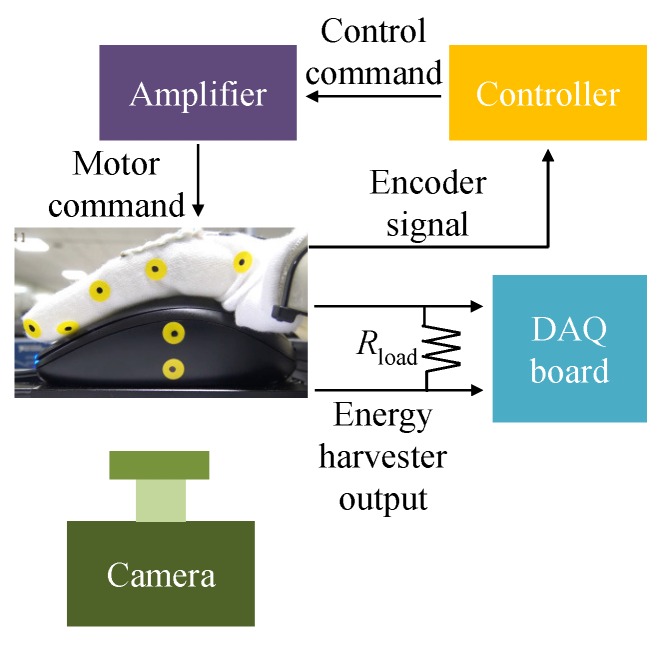
Experimental setup for a robot finger.

In the second phase of the experimental study, we use a robot finger [37,38] for studying the energy harvesting. The prosthetic robot finger is designed to generate finger motions such as pinch grasping motion, see Figure 4. Basically, the prosthetic finger system contains underactuated mechanism, which is a linkage mechanism, in order to implement compliant motion similar to human finger motion with respect to unknown grasping objects. The benefit of the underactuated mechanism is the reduction of the number of actuator in the robot system. Specifically, only one joint is actuated by a motor, and the other two joints are passively rotated by transferred power with the linkage mechanism. Therefore, the robot finger is able to generate human-like compliant motion. FAULHABER 1717T024SR DC-micro motor, controlled by MAXON LSC 30/2 servo amplifier and PC104 main controller, is used to actuate the robot finger and to receive the encoder signal with 1000Hz. The main controller is programmable using MATLAB simulink. During the experiments, the robot finger wears also the glove including the energy harvester. Other experimental setup is same to Section 3.1. The experimental setup is displayed in Figure 5.

## 4. Results

### 4.1. Motion Trajectory of Human Finger

**Figure 6:**
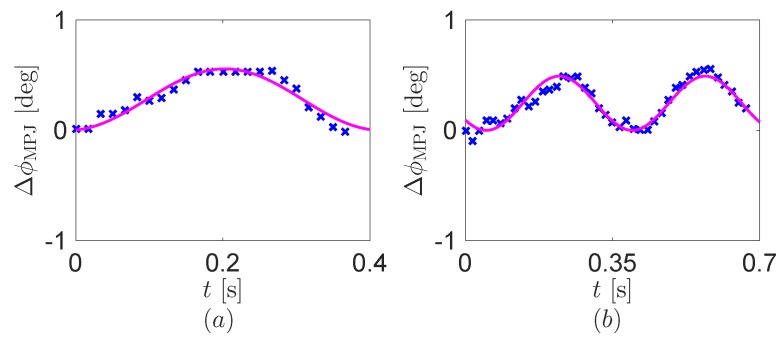
The angle $\phi_{\mathrm{MPJ}}$ of the human index finger. (**a**) One click and (**b**) double click. Blue crosses and purple lines are the experimental data and its fundamental harmonics, respectively.

We capture the motion trajectory of the human index finger during a mouse one click and a double click. Specifically, the angles of the MP joint $\phi_{\mathrm{MPJ}}$ are obtained by using the recoding camera data, see Figure 6. The human index finger during the mouse clicks has the tiny movements under $1^{\circ }$ and approximately the frequency of 2–3Hz. Additionally, to formulate the angle trajectory, we perform Fourier cosine series fitting by using the built-in function ‘fit’ in MATLAB. Figure 6 displays the fitting results including the fundamental harmonic of the angle. The coefficients of determination R-squared of the fitting are 0.9221 at the one click and 0.9029 at the double click. Therefore, the mouse click motions of the human index finger can be presented by a cosine function.

### 4.2. Operation of Robot Finger

**Figure 7:**
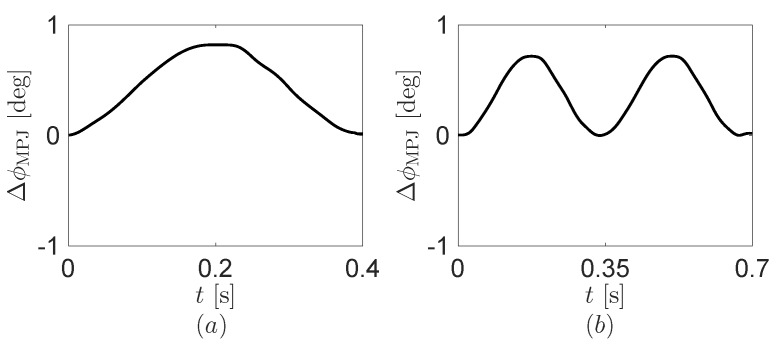
Encoder signal from the robot finger. (**a**) One click and (**b**) double click.

The fitting results of the motion trajectory in Figure 6 are used for the operation of the motor in the robot finger. In the robot finger, we use the amplitude increased by 50% due to the friction at the connection between the motor and the robot finger. The angle $\phi_{\mathrm{MPJ}}$ programmed in the robot finger is a(1-cos(2πft)) where $a=0.4179^{\circ }$ and f=2.353 at the one click, and $a=0.3683^{\circ }$ and f=2.877 at the double click. For the double click, we use two periods of the cosine function. Figure 7 displays the encoder signal from the robot finger. We comment that the angle from the encoder signal can be different with the real angle because of the friction at the connection in the robot finger.

**Figure 8:**
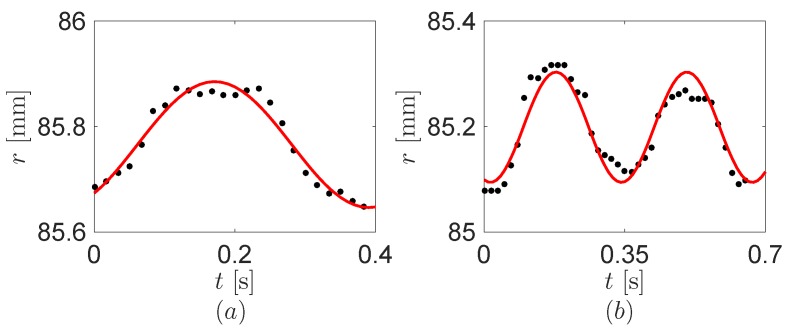
The distance *r* between the tip and the metacarpophalangeal (MP) joint of the robot finger. (**a**) One click and (**b**) double click. Black dots and red lines are the experimental data and its fundamental harmonics, respectively.

Figure 8 shows the distance *r* between the tip and the MP joint of the robot finger using the recoding camera data. To use the distance in the model of Section 2, we also fit the distance to the Fourier cosine series about the fundamental harmonic through MATLAB.

### 4.3. Open-Circuit Voltage from Robot Finger

**Figure 9:**
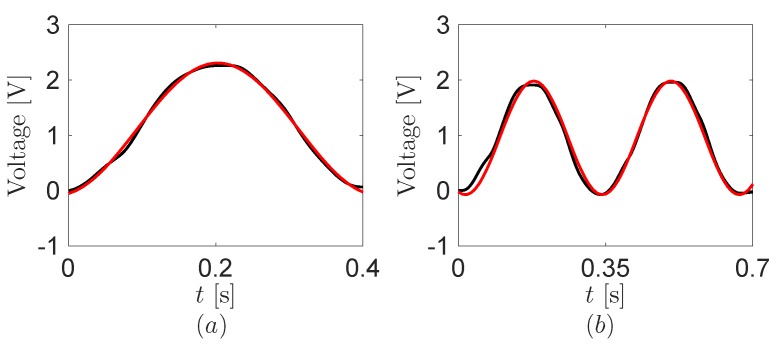
Open-circuit voltage output from the energy harvester. (**a**) One click and (**b**) double click. Black and red lines are the experimental data and its fundamental harmonics, respectively.

To obtain the coupling coefficient *θ* of the energy harvester, we perform an experiment about its electrical responses at the open-circuited electrodes, that is, $R_{load}=\infty$ in Figure 5. In practice, the electrodes of the the energy harvester are shunted with the large input impedance of the DAQ board to record the voltage output. Such impedance is over one thousand times larger than the matching impedance of the piezoelectric material in the frequency of the mouse click motions, based on the measured capacitance. Figure 9 displays the open-circuit voltage output from the energy harvester during the both mouse click motions. The electrical responses are dominated by the fundamental harmonic at the frequency of the mouse click motions. We obtain θ=-158nJ/V using the one click data of Figure 8 and Figure 9 with the model in Section 2.

### 4.4. Energy Harvested from Robot Finger

**Figure 10:**
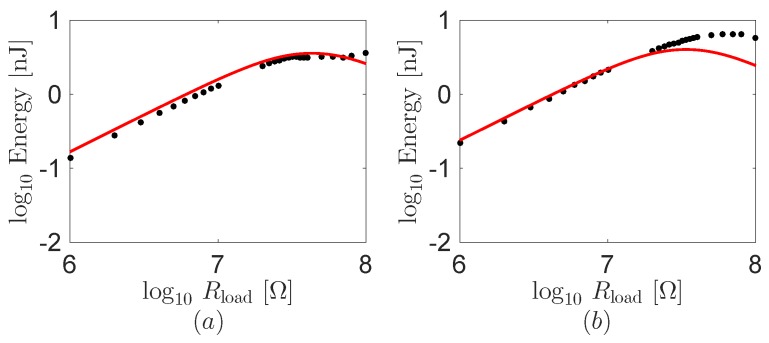
Theoretical predictions and experimental results on energy transferred to the load resistance as a function of the resistance using the robot finger. (**a**) One click and (**b**) double click. Black dots and red lines are the experimental results and the theoretical predictions, respectively.

The overall modeling framework in Section 2 allows for predicting the energy harvested at the load resistance $R_{\mathrm{load}}$. Figure 10 illustrates the computation results of the harvested energy as a function of the load resistance. Therein, the harvested energy from the experiments is also overlapped. The comparison between the theoretical predictions and experimental results demonstrates that the model is fairly accurate in predicting the harvested energy varying the load resistance. The maximum harvested energy is in the range of 1–10nJ at the both mouse clicks. Experimental results indicate that the harvested energy is maximized for load resistances on the range of 30–70MΩ, which correspond to the matching impedance 1/(2πfC) of the piezoelectric material. Some discrepancy between the theoretical predictions and experimental results is likely to be attributed to the change of the electromechanical property by the movement and the initial curve of the energy harvester in the pocket during the experiments. Additionally, half harmonic frequency may be relevant in the double click experiments. It makes the maximum point move into higher one (1/(πfC)).

### 4.5. Energy Harvested from Human Finger

**Figure 11:**
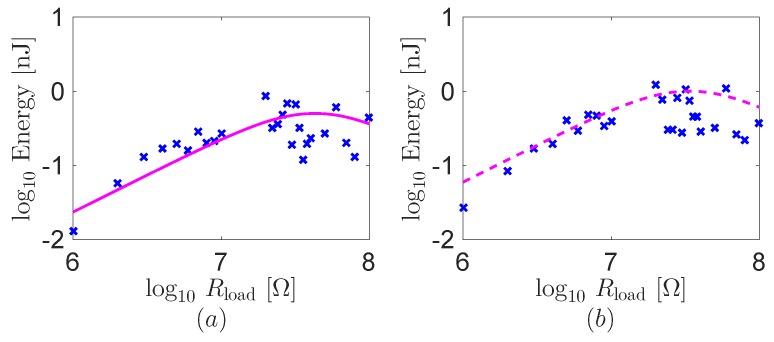
Theoretical predictions and experimental results on energy transferred to the load resistance as a function of the resistance using the human index finger. (**a**) One click and (**b**) double click. Blue crosses and purple lines are the experimental results and the theoretical predictions, respectively.

We adapt the overall modeling framework for the harvested energy to the human finger. To reduce the effect of the variation of the human motion, we use the average value of the five repeated experiments at the each load resistance. Figure 11 displays the theoretical predictions and experimental results of the energy harvested from the human finger during the both mouse clicks. Although the human motions are not perfectly same for all trials unlike the robot, the theoretical predictions are also in good agreement with the experimental results in anticipating the trend of the energy as a function of the load resistance. The maximum energy harvested from the human finger is on the order of 1nJ at the both mouse clicks. We note that the lower energy level of the human finger than the robot results from the structural difference by different size ($L_{1}$, $L_{2}$, and $L_{3}$)of the fingers. In other words, the different finger size of the human finger makes smaller $\Delta \phi_{\mathrm{PIJ}}$ than the robot.

## 5. Conclusions

In this paper, we have analyzed energy harvesting from mouse click motions using a piezoelectric material. We have developed a mathematical model for the electromechanical behavior of the system to predict the energy harvested from the finger motion during the mouse clicks. The finger motion has been described as a three degree of freedom mechanism consisting of three links in a plane. The electromechanical coupling of the piezoelectric material has been considered a relationship between its stored charge and rotation angle. To validate the modeling framework, we have conducted experiments about harvested energy at the varying load resistances. As the test subject, we have used a robot finger and a human index finger. The energy harvester has been attached to the both fingers by using a glove with a pocket.

Theoretical predictions of energy harvesting have been found to be in good agreement with experimental results for the both human and robot fingers, corroborating the validity of the proposed modeling approach. Our results indicate that energy harvesting is optimized when the load resistance matches the impedance of the piezoelectric material for the fundamental harmonic, and the maximum harvested energy is in the range of 1–10nJ. We anticipate that the energy level can be improved by using multiple layers or alternative smart materials with high efficiency. We expect that the experimental results and the modeling framework presented in this study can find application in the design of self-powered mouse or wearable devices through energy harvesting from human motions.
